# IncN-R plasmid co-integration contributing to extensive drug resistance in *Escherichia coli* isolated from a canine prostatic abscess

**DOI:** 10.1128/spectrum.00790-25

**Published:** 2025-07-30

**Authors:** Parinya Sroithongkham, Chavin Leelapsawas, Rusmin Indra, Sunchai Payungporn, Komkiew Pinpimai, Suppawiwat Ponglowhapan, Pattrarat Chanchaithong

**Affiliations:** 1 Division of Pathobiology, Faculty of Veterinary Medicine, Khon Kaen University 737460, Khon Kaen, Thailand; 2 Department of Veterinary Microbiology, Faculty of Veterinary Science, Chulalongkorn University 71651, Bangkok, Thailand; 3 Center of Excellence in Systems Microbiology, Department of Biochemistry, Faculty of Medicine, Chulalongkorn University 65103, Bangkok, Thailand; 4 Aquatic Resources Research Institute, Chulalongkorn University 627646, Bangkok, Thailand; 5 Department of Obstetrics, Gynaecology and Reproduction, Faculty of Veterinary Science, Chulalongkorn University 627493, Bangkok, Thailand; 6 Research Unit in Microbial Food Safety and Antimicrobial Resistance, Chulalongkorn University 26683, Bangkok, Thailand; Universita degli Studi dell'Insubria, Varese, Italy

**Keywords:** *bla*_CTX-M-55_, dogs, extended-spectrum β-lactamases, multi-replicon plasmid

## Abstract

The emergence of multidrug-resistant (MDR) plasmids poses a significant global public health threat. This study characterized a novel multi-replicon IncN-R MDR plasmid pCUVET19-1426.1, harboring *bla*_CTX-M-55_ and 12 additional antimicrobial resistance genes (ARGs) in a newly identified *Escherichia coli* sequence type 13037, isolated from a canine prostatic abscess. Using antimicrobial resistance (AMR) profiling and genomic analysis, we elucidated the characteristics, evolution, and transferability of plasmid pCUVET19-1426.1, which contributed to the extensively drug-resistant phenotype of the bacterial strain. The plasmid is genetically related to IncN plasmids found in Enterobacterales from humans and animals in Thailand, suggesting plasmid circulation and evolution. Multiple IS*26* elements enhanced the plasmid plasticity for co-integration and acquisition of ARGs, underscoring its significant clinical and public health impact. Our findings emphasize the importance of genomic insights in understanding plasmid evolution and its role in AMR development in *E. coli,* causing opportunistic infections.

**IMPORTANCE:**

This study highlights the significance of plasmid evolution to generate a new multi-replicon IncN-R plasmid encoding an extended-spectrum β-lactamase and multidrug resistance (MDR) in a new *Escherichia coli* sequence type 13037 from a canine prostatic abscess. We provide insights into the genetic mechanisms facilitating the spread and persistence of resistance genes, driving the emergence of an extensively drug-resistant phenotype. The presence of an MDR plasmid in the clinical *E. coli* isolate critically limited antimicrobial options for extraintestinal infections in small animal veterinary medicine. The plasmid’s genetic relatedness underscores the interconnectedness of human and animal health, emphasizing the importance of a One Health approach. These findings emphasize the need to enhance genomic surveillance to monitor the evolution of bacterial plasmids and their role in antimicrobial resistance development in both human and animal health.

## OBSERVATION

Extended-spectrum β-lactamase (ESBL)-producing *Escherichia coli*, which hydrolyze third-generation cephalosporins (3GCs) and are associated with multidrug resistance, pose a significant treatment challenge in *E. coli,* causing extraintestinal infections in human and veterinary medicine (1, 2). Of particular concern is the increasing prevalence of the *bla*_CTX-M-55_ carriage, which was previously found primarily in commensal isolates, as well as in isolates from livestock and the environment but is now spreading among clinical strains (3, 4).

Conjugative plasmids play a crucial role in the horizontal transfer of antimicrobial resistance genes (ARGs), with incompatibility group N (IncN) plasmids being broad-host-range, highly transferable, and capable of recombining into hybrid plasmids. In this study, we characterized a novel multi-replicon IncN-R plasmid carrying *bla*_CTX-M-55_ and 12 additional ARGs in a newly identified *E. coli* sequence type (ST) from a canine prostatic abscess.

The *E. coli* strain CUVET19-1426 was isolated from the purulent prostatic exudate of a 15-year-old mixed-breed intact male dog diagnosed with a prostatic abscess at the Small Animal Teaching Hospital of Chulalongkorn University, Thailand, in 2019. Bacterial identification was performed using Microflex MALDI-TOF MS (Bruker Daltonics, Germany). The *E. coli* strain exhibited 3GC resistance and an extensively drug-resistant (XDR) phenotype, as determined by broth microdilution using a Sensititre™ COMPGN1F plate (Thermo Fisher Scientific, USA; Table S1). Additionally, ESBL production was confirmed using a combination disk test (5).

The whole genome of *E. coli* CUVET19-1426 was sequenced using the Illumina NextSeq 550 and Oxford Nanopore Technologies platforms. Hybrid assembly was performed with Unicycler v.0.4.8, resulting in a complete circular chromosome of 4,836,704 bp with a GC content of 50.84% and four plasmids (Table 1) (6). Gene annotation was performed using the NCBI Prokaryotic Genome Annotation Pipeline (7). Phylogrouping, multilocus sequence typing (MLST), and core genome MLST (cgMLST) were performed using Enterobase (http://enterobase.warwick.ac.uk). The *E. coli* strain belonged to phylogroup A and was identified as a new ST13037 with an allelic profile of 9-41-1-8-8-8-6. cgMLST analysis classified it as cgST191738. SerotypeFinder v2.0 and the Virulence Factor Database identified the strain as serotype O9:H4, carrying 37 virulence genes encoding nine virulence factors (8, 9) (Table S2). Acquired ARGs and quinolone resistance-determining region (QRDR) mutations were detected using ResFinder v.4.1 and NCBI Antimicrobial Resistance (AMR) Finder (10, 11). Plasmid replicons and mobile genetic elements were identified using PlasmidFinder v.2.1 and MobileElementFinder v.1.0.3 (12, 13). Plasmid transferability to the rifampicin-resistant *E. coli* K-12 MG1655 recipient strain by conjugation was assessed by a filter mating assay (14). AMR phenotypes of transconjugants were examined using minimum inhibitory concentration determination.

**TABLE 1:** Characteristics of plasmids carried by extensively drug-resistant *E. coli* strain CUVET19-1426^*a*^

Plasmid name	Size (bp)	Incompatibility group	Plasmid sequence type	Acquired antimicrobial resistance gene(s)
pCUVET19-1426.1	123,620	N, R	ST1 (IncN)	*bla*_CTX-M-55_, *bla*_TEM-1_ (x2), *qnrS13*, *aadA1*, *aadA2*, *tet*(A), *dfrA12*, *sul2*, *sul3*, *floR*, *cmlA1,* and *mef*(B)
pCUVET19-1426.2	69,932	FII	F33:A-:B-	*bla*_TEM-1_ and *rmtB*
pCUVET19-1426.3	43,410	X1	–	*tet*(M)
pCUVET19-1426.4	6,710	Col156	–	–

^*a*^ “–” indicates none.

Three amino acid substitutions in QRDRs were observed: *gyrA*:pS83L, *gyrA*:pD87N, and *parC*:pS80I; however, no acquired ARGs were identified in the chromosome. Of the four plasmids, three—pCUVET19-1426.1, pCUVET19-1426.2, and pCUVET19-1426.3—harbored ARGs, while pCUVET19-1426.4 was a cryptic plasmid (Table 1). The ARGs were associated with extensive drug resistance across multiple antimicrobial classes (Table 2). The multi-replicon IncN-R plasmid pCUVET19-1426.1 carried *bla*_CTX-M-55_ and 12 additional ARGs. IncN plasmids are predominantly found in commensal *E. coli* isolated from humans, animals, and the environment, but they are rarely detected in *E. coli* from veterinary clinical specimens (15, 16).

**TABLE 2:** Acquired antimicrobial resistance genes and mechanisms associated with the antimicrobial resistance phenotype of *E. coli* strain CUVET19-1426

Acquired ARG	Resistance mechanism	Antimicrobial resistance phenotype
β-lactams		
*bla*_TEM-1_	TEM-1 β-lactamase	Ampicillin
*bla*_CTX-M-55_	CTX-M-55 extended-spectrum β-lactamase	Ampicillin, cephalexin, cefazolin, cefpodoxime, cefovecin, and ceftazidime
Fluoroquinolones		
*qnrS13*	Plasmid-mediated quinolone resistance QnrS13 protein	Enrofloxacin, marbofloxacin, pradofloxacin, and orbifloxacin
Aminoglycosides		
*aadA1* and *aadA2*	Aminoglycoside adenylyltransferase	Streptomycin^*a*^
*rmtB*	Ribosomal methyltransferase	Gentamicin and amikacin
Tetracyclines		
*tet*(A)	Tetracycline efflux protein	Tetracycline and doxycycline
*tet*(M)	Ribosomal protective protein	Tetracycline and doxycycline
Macrolides		
*mef*(B)	Macrolide efflux protein	Erythromycin^*a*^
Dihydrofolate reductase inhibitors		
*dfrA12*	Alternative dihydrofolate reductase	Trimethoprim
Sulfonamides		
*sul2* and *sul3*	Alternative dihydropteroate synthase	Sulfamethoxazole
Phenicols		
*cmlA1*	Chloramphenicol exporter	Chloramphenicol
*floR*	Florfenicol/chloramphenicol exporter	Chloramphenicol

^*a*^ Streptomycin and erythromycin resistance were not examined in this study.

**Fig 1:**
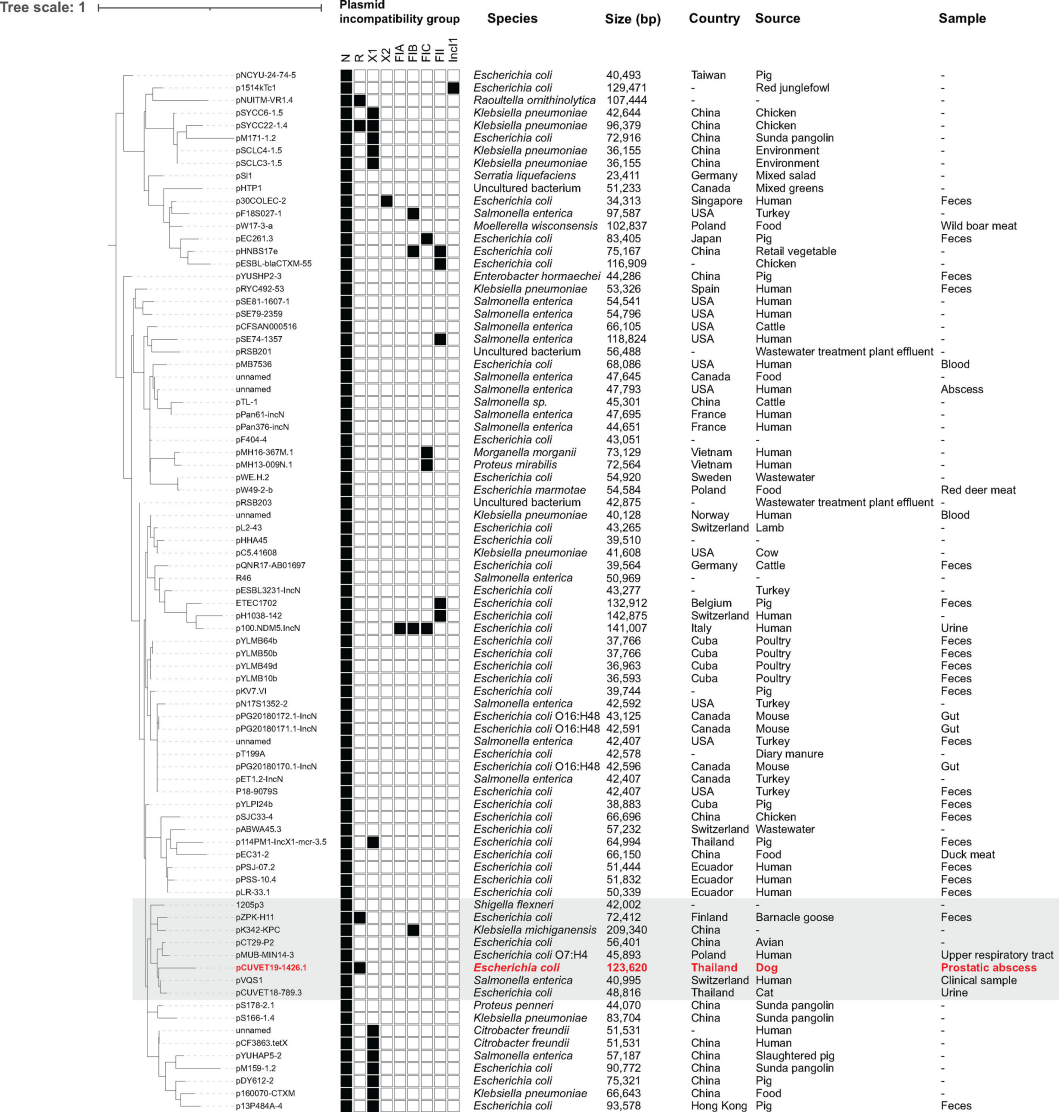
Phylogenetic tree of 83 IncN plasmids (sequence type 1) carrying antimicrobial resistance gene(s), retrieved from the NCBI database, generated using orthologous protein comparison with Roary. The tree is drawn to scale, with the cluster containing pCUVET19-1426.1 (GenBank accession no. CP115366) and closely related plasmids highlighted in gray. GenBank accession numbers and references for each plasmid are provided in Table S3.

The pCUVET19-1426.1 was identified as IncN ST1 by using the Plasmid MLST database (https://pubmlst.org/organisms/plasmid-mlst). Phylogenetic analysis of 82 complete IncN (ST1) resistance plasmid sequences from the NCBI database (Table S3) was conducted alongside pCUVET19-1426.1. The phylogenetic tree, based on gene presence/absence data from Roary (17), revealed that pCUVET19-1426.1 was closely related to pCUVET18-789.3 (GenBank accession no. CP115315) from *E. coli* ST641 isolated from a feline urinary tract infection in Thailand and pVQS1 (GenBank accession no. JQ609357) from *Salmonella enterica* in a Swiss patient with a travel history to Thailand (Fig. 1) (18). However, neither pCUVET18-789.3 nor pVQS1 contained *bla*_CTX-M-55_.

**Fig 2:**
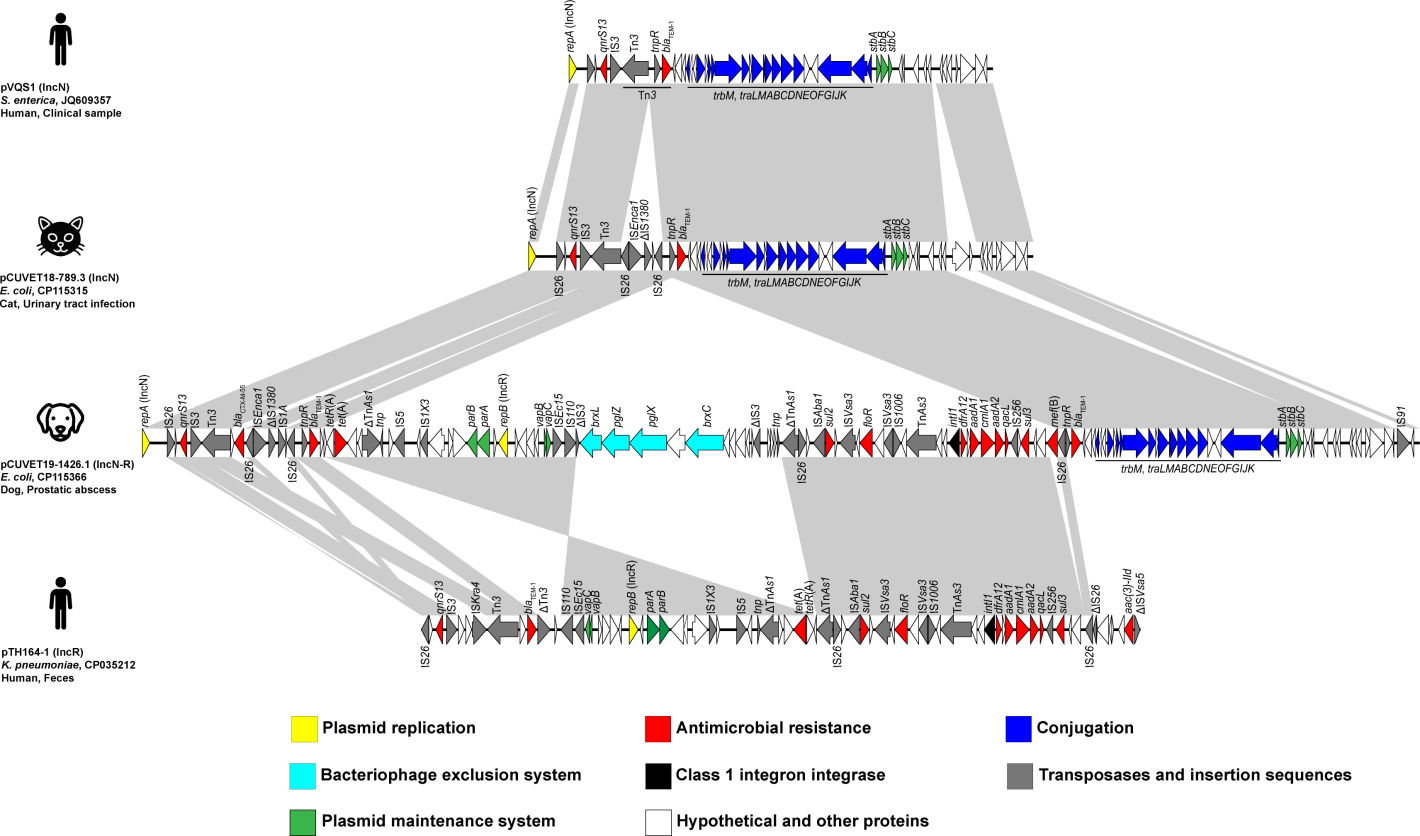
Schematic representation and sequence comparison of the *bla*_CTX-M-55_-carrying IncN-R pCUVET19-1426.1 plasmid (GenBank accession no. CP115366) found in *E. coli* isolated from a canine prostatic abscess in Thailand. The multi-replicon IncN-R pCUVET19-1426.1 plasmid sequence is compared with the IncN plasmids: pVQS1 (GenBank accession no. JQ609357) in *S. enterica* isolated from a human patient in Switzerland, and pCUVET18-789.3 (GenBank accession no. CP115315) in *E. coli* isolated from cat urine in Thailand, and IncR plasmid: pTH164-1 (GenBank accession no. CP035212) in *K. pneumoniae* isolated from human stool in Thailand. The arrows and the colors of each arrow indicate the orientation and function of the genes as given in the legend below. The symbol “∆” indicates truncation. The gray shading indicates more than 95% sequence similarity.

The pCUVET19-1426.1 encoded 136 genes and was divided into five regions: two IncN plasmid backbone regions located at both extremities, two IncR plasmid regions in between, and a central region containing bacteriophage exclusion (BREX) loci (Fig. 2). The IncN plasmid regions included the IncN replication initiation gene (*repA*), genes encoding conjugation module (*traLMABCDNEOFGIJK*), and a toxin/antitoxin system (*stbABC*). The variable region contained insertion sequence (IS) elements and ARGs. These regions shared homology with the IncN plasmids pCUVET18-789.3 and pVQS1. The IncR regions contained the replication initiation gene (*repB*), genes encoding plasmid maintenance system, and toxin/antitoxin system (*vapBC*). The multi-replicon IncN-R plasmid pCUVET19-1426.1 also shared 99.89% sequence identity with 53% coverage to the IncR plasmid pTH164-1 (GenBank accession no. CP035212) in *Klebsiella pneumoniae* ST873 strain TH164, which was isolated from human stool in Thailand. Additionally, the IncR plasmid backbone and variable regions of pCUVET19-1426.1 were interrupted by the insertion of the BREX system. *bla*_CTX-M-55_, *qnrS13*, and *bla*_TEM-1_ genes were located downstream of *repA* in the IncN variable region of pCUVET19-1426.1. However, only *qnrS13* and *bla*_TEM-1_ were found in the IncN plasmids pCUVET18-789.3 and pQVS1. The *tet*(A) gene was located in the 5' region of the IncR plasmid backbone, which represented an inverted region compared to the IncR plasmid pTH164-1 (Fig. 2). Most ARGs were located in the IncR variable region, which contained a class 1 integron associated with *dfrA12*, *aadA1*, *cmlA1*, and *aadA2*, as well as *sul2*, *sul3*, *floR*, and *mef*(B). An additional copy of *bla*_TEM-1_ was observed downstream of the IncR variable region, resulting in the IncR region being flanked by two copies of *bla*_TEM-1_. Based on structural comparison and phylogenetic analysis, pCUVET19-1426.1 likely evolved from a local IncN resistance plasmid in Thailand before acquiring *bla*_CTX-M-55_. Additionally, recombination with the IncR plasmid harboring multiple ARGs further promoted XDR properties. Altogether, co-integration could provide selective advantages to plasmids and bacteria by increasing the number of ARGs, thus conferring resistance to multiple antimicrobials.

The pCUVET19-1426.1 contained copies of the IS*26* element, which could play roles in *bla*_CTX-M-55_ insertion and plasmid co-integration. *bla*_CTX-M_ genes are typically preceded by an IS*26* element that facilitates gene mobilization and contains the gene promoter (19). The co-integration between these IncN and IncR plasmids likely resulted from homologous recombination or conservative transposition, mediated by IS*26*, rather than replicative transposition (20, 21). However, whether the integration of *bla*_CTX-M-55_ into the IncN plasmid occurred before or after recombination remains unclear. Therefore, IS*26*-mediated transposition of *bla*_CTX-M-55_ contributed to AMR development through plasmid co-integration.

Multiple ARGs were located on variable regions of pCUVET19-1426.1, associated with various IS elements. The IncR variable region contained a higher number of ARGs due to the presence of a class 1 integron. In addition to AMR development, plasmid co-integration enhances maintenance and transferability. The pCUVET19-1426.1 exhibited a high conjugation frequency of 3 × 10⁻⁴, and the transconjugant exhibited AMR phenotypes consistent with the ARGs encoded on the plasmid (Table 1). Its IncN backbone of pCUVET19-1426.1 harbored conjugative modules, enabling broad-host-range and high transferability among Enterobacterales (18). The ParAB partitioning system in the IncR backbone supports plasmid segregation (22). Although neither progenitor contained a type 1 BREX system, an additional insertion in the IncR backbone may defend against phages by restricting foreign DNA (23). Thus, plasmid recombination enhances stability, transferability, and bacterial survival.

In conclusion, the novel IncN-R plasmid harboring *bla*_CTX-M-55_ in a clinical canine *E. coli* isolate is genetically related to ancestral IncN plasmids found in human Enterobacterales from Thailand. Moreover, the occurrence of this plasmid in a novel *E. coli* lineage highlights the ongoing evolution and the potential for widespread dissemination of resistance determinants. Although *E. coli* phylogroup A is primarily associated with commensal strains in the intestine of humans and animals, it can translocate from the intestinal tract and cause prostate infections (24). Thus, intestinal carriage of XDR *E. coli* increases the risk of extraintestinal infections caused by resistant strains, limiting antimicrobial options and leading to therapeutic challenges. These findings emphasize the importance of genomic surveillance in monitoring the plasmid evolution in *E. coli* from various sources.

## Data Availability

The genome sequences of *E. coli* CUVET19-1426 are deposited in the NCBI database under BioProject accession number PRJNA914526.

## SUPPLEMENTAL MATERIAL

Table S1 to S3 spectrum.00790-25-s0001.pdf Table S1 Antimicrobial resistance phenotype. Table S2 Genes encoding virulence factors. Table S3 Reference sequences of IncN resistance plasmids.

ASM does not own the copyrights to Supplemental Material that may be linked to, or accessed through, an article. The authors have granted ASM a non-exclusive, world-wide license to publish the Supplemental Material files. Please contact the corresponding author directly for reuse.
