# Supplementary material for: IncN-R plasmid co-integration contributing to extensive drug resistance in Escherichia coli isolated from a canine prostatic abscess
Source: Microbiol Spectr. 2025 Jul 30;13(9):e00790-25. doi: 10.1128/spectrum.00790-25 (PMC12403796; doi:10.1128/spectrum.00790-25)
Supplement: Table S1 to S3 — Table S1 Antimicrobial resistance phenotype. Table S2 Genes encoding virulence factors. Table S3 Reference sequences of IncN resistance plasmids. [file spectrum.00790-25-s0001.pdf]

Supplemental material

IncN-R plasmid co-integration contributing to extensive drug resistance in *Escherichia coli* isolated from a canine prostatic abscess

Parinya Sroithongkham, Chavin Leelapsawas, Rusmin Indra, Sunchai Payungporn,
Komkiew Pinpimai, Suppawiwat Ponglowhapan, Pattrarat Chanchaithong

Table S1 Antimicrobial resistance phenotypes based on minimum inhibitory concentrations (MICs) of the extensively drug-resistant *Escherichia coli* strain CUVET19-1426, *E. coli* recipient strain K12 MG1655, and the corresponding transconjugant.

**Antimicrobial**	**MIC in µg/mL (Interpretation*)**		
	**CUVET19-1426**	**K12 MG1655**	**Transconjugant**
Ampicillin	>8 (R)	4 (S)	>8 (R)
Amoxicilin/clavulanic acid	>8/4 (R)	8/4 (S)	>8/4 (R)
Piperacillin/tazobactam	≤8/4 (S)	≤8/4 (S)	≤8/4 (S)
Cephalexin	>16 (R)	16 (S)	>16 (R)
Cefazolin	>32 (R)	4 (S)	>32 (R)
Cefpodoxime	>8 (R)	≤1 (S)	>8 (R)
Cefovecin	>8 (R)	1 (S)	>8 (R)
Ceftazidime	16 (R)	≤4 (S)	8 (I)
Imipenem	≤1 (S)	≤1 (S)	≤1 (S)
Amikacin	>32 (R)	≤4 (S)	≤ 4 (S)
Gentamicin	>8 (R)	0.5 (S)	0.5 (S)
Enrofloxacin	>4 (R)	≤0.12 (S)	2 (R)
Marbofloxacin	>4 (R)	≤0.12 (S)	2 (R)
Pradofloxacin	>2 (R)	≤0.25 (S)	0.5 (I)
Orbifloxacin	>8 (R)	≤1 (S)	>8 (R)
Doxycycline	>8 (R)	4 (S)	>8 (R)
Tetracycline	>16 (R)	≤4 (S)	>16 (R)
Chloramphenicol	>32 (R)	8 (S)	>32 (R)
Sulfamethoxazole/trimethoprim	>76/4 (R)	≤9.5/0.5 (S)	>76/4 (R)

*Antimicrobial susceptibility was interpreted according to the MIC breakpoints for veterinary isolates from the Clinical and Laboratory Standards Institute (1).

Table S2. Genes encoding virulence factors identified in the genome of *Escherichia coli* strain CUVET19-1426.

**Virulence**	**Virulence factor**	**Virulence gene**
Adhesins	Curli fimbria	*csgBDFG*
	E. coli common pilus	*ecpABCER*
	Factor adherence *E. coli*	*fdeC*
	Type 1 fimbria	*fimDFGH*
Toxin production	Serine protease autotransporters of *Enterobacteriaceae*	*espL1*,*4*; *espR1*,*4*; and *espX1*,*4*,*5*
Iron acquisition system	Enterobactin	*entABCDEFS*
	Ferrienterobactin	*fepABCDG* and *fes*
Secretion system	Type II secretion system	*gspLM*
Protectin	Outer membrane protein A	*ompA*

Table S3. Reference sequences of IncN resistance plasmids collected from the National Center for Biotechnology Information (NCBI) database included for plasmid phylogenetic analysis in this study.

Plasmid name	Bacterial species	GenBank accession no.	Size (bp)	Year	Country	Resistance gene	Reference
pPan376-incN	*Salmonella enterica*	OR797034.1	44,651	1966	France	*bla*$_{\text{TEM-1}}$, and *tet*(A)	–
pPan61-incN	*Salmonella enterica*	OR797032.1	47,695	1966	France	*bla*$_{\text{TEM-1}}$, and *tet*(A)	–
pSE74-1357	*Salmonella enterica*	CP018643.1	118,824	1974	USA	*bla*$_{\text{TEM-1}}$, *tet*(A), and *strAB*	–
pSE79-2359	*Salmonella enterica*	CP018646.1	54,796	1979	USA	*bla*$_{\text{TEM-1}}$, and *strAB*	–
pSE81-1607-1	*Salmonella enterica*	CP018649.1	54,541	1981	USA	*bla*$_{\text{TEM-1}}$, and *strAB*	–
pCFSAN000516	*Salmonella enterica*	CP074230.1	66,105	1986	USA	*bla*$_{\text{TEM-1}}$, *tet*(A), *strAB*, *aph(3')-IIa*, and *aph(6)-Ic*	–
ETEC1702	*Escherichia coli*	CP122931.1	132,912	2012	Belgium	*bla*$_{\text{CTX-M-1}}$, *bla*$_{\text{TEM-1}}$, *aadA1*, and *sul1*	–
pEC261_3	*Escherichia coli*	AP027420.1	83,405	2012	Japan	*bla*$_{\text{TEM-1}}$, *strAB*, *aadA1*, *aadA2*, and *dfrA12*	–
p13P484A-4	*Escherichia coli*	CP019284.1	93,578	2013	Hong Kong	*bla*$_{\text{TEM-106}}$, *strAB*, *aac(3)-IId*, and *catA2*	–
pH1038-142	*Escherichia coli*	KJ484634.1	142,875	2013	Switzerland	*bla*$_{\text{CTX-M-1}}$, *bla*$_{\text{TEM-1}}$, *tet*(A), *strAB*, *aadA1*, *dfrA1*, *sul3*, *catA1*, *mef*(B), and *mph*(A)	–
pL2-43	*Escherichia coli*	KJ484641.1	43,265	2013	Switzerland	*bla*$_{\text{CTX-M-1}}$, and *mph*(A)	–
pYLMB50b	*Escherichia coli*	CP073951.1	37,766	2013	Cuba	*bla*$_{\text{CTX-M-32}}$	–
unnamed1	*Salmonella enterica*	CP026053.1	47,793	2013	USA	*bla*$_{\text{TEM-1}}$, and *tet*(A)	–
pCT29-P2	*Escherichia coli*	CP032075.1	56,401	2014	China	*bla*$_{\text{TEM-1}}$ *oqxA*, *oqxB*, and *qnrS1*,	–
pYLMB49d	*Escherichia coli*	CP073957.1	36,963	2014	Cuba	*bla*$_{\text{CTX-M-32}}$	–
unnamed2	*Salmonella enterica*	CP133567.1	47,645	2014	Canada	*bla*$_{\text{TEM-1}}$, and *tet*(A)	–
pYLMB10b	*Escherichia coli*	CP073990.1	36,593	2015	Cuba	*bla*$_{\text{CTX-M-32}}$	–
pYLMB64b	*Escherichia coli*	CP073931.1	37,766	2015	Cuba	*bla*$_{\text{CTX-M-32}}$	–
unnamed_2	*Klebsiella pneumoniae*	CP073790.1	40,128	2015	Norway	*bla*$_{\text{CTX-M-1}}$	–
pET1.2-IncN	*Salmonella enterica*	CP043224.1	42,407	2016	Canada	*bla*$_{\text{CTX-M-1}}$	–
pF18S027-1	*Salmonella enterica*	CP082433.1	97,587	2016	USA	*bla*$_{\text{HERA-3}}$, *strAB*, *aadA1*, *aac(3)-Via*, and *sul1*	–
pMUB-MIN14-3	*Escherichia coli*	CP069649.1	45,893	2016	Poland	*bla*$_{\text{TEM-1}}$, and *qnrS1*	–
pYUHAP5-2	*Salmonella enterica*	CP060136.1	57,187	2016	China	*bla*$_{\text{TEM-1}}$, *aph(3')-IIa*, *aadA22*, *rmtB*, and *lnu*(F)	–
pEC31-2	*Escherichia coli*	CP056038.1	66,150	2017	China	*bla*$_{\text{TEM-106}}$, *qepA*, *qnrS1*, *tet*(A), and *aadA1*	–
pN17S1352-2	*Salmonella enterica*	CP082632.1	42,592	2017	USA	*bla*$_{\text{CTX-M-1}}$	–
pNCYU-24-74-5	*Escherichia coli*	CP042643.1	40,493	2017	Taiwan	*aac(3)-IId*	–

Plasmid name	Bacterial species	GenBank accession no.	Size (bp)	Year	Country	Resistance gene	Reference
pQNR17-AB01697	*Escherichia coli*	CP081648.1	39,564	2017	Germany	*bla*$_{\text{CTX-M-1}}$, *qnrB20*, *aadA1*, *dfrA25*, and *sul1*	–
pSJC33-4	*Escherichia coli*	CP080259.1	66,696	2017	China	*bla*$_{\text{TEM-1}}$, *strAB*, *tet*(A), *sul2*, and *cfr*	–
p114PM1-IncX1-mcr-3.5	*Escherichia coli*	CP064008.1	64,994	2018	Thailand	*mcr-3*	–
pABWA45_3	*Escherichia coli*	CP022157.1	57,232	2016	Switzerland	*bla*$_{\text{TEM-1}}$, and *rmtB*	–
pCF3863_tetX	*Citrobacter freundii*	CP059850.1	51,531	2018	China	*tet*(X)	–
pCUVET18-789.3	*Escherichia coli*	CP115315.1	48,816	2018	Thailand	*bla*$_{\text{TEM-1B}}$, and *qnrS1*	–
pHTP1	uncultured bacterium	MH339998.1	51,233	2018	Canada	*qnrB5*, *tet*(A), *aadA1*, *aadA2*, *aph(3')-Ia*, *dfrA12*, *sul1*, and *mph*(A)	–
pM159-1.2	*Escherichia coli*	CP058721.1	90,772	2018	China	*bla*$_{\text{CTX-M-65}}$, *bla*$_{\text{TEM-1}}$, *bla*$_{\text{TEM-104}}$, *bla*$_{\text{TEM-141}}$, *bla*$_{\text{OXA-10}}$, *qepA*, *tet*(A), *aadA1*, *aph(3')-IIa*, *dfrA14*, *cmlA1*, *fosA*, and *ARR-3*	–
pM171-1.2	*Escherichia coli*	CP101668.1	72,916	2018	China	*bla*$_{\text{CTX-M-65}}$, *bla*$_{\text{TEM-1}}$, *bla*$_{\text{OXA-10}}$, *qepA*, *tet*(A), *aadA1*, *aph(3')-IIa*, *dfrA14*, *cmlA1*, and *ARR-3*	–
pMB7536_2	*Escherichia coli*	CP103598.1	68,086	2018	USA	*bla*$_{\text{TEM-10}}$, *tet*(A), and *floR*	–
pS178-2.1	*Proteus penneri*	CP059691.1	44,070	2018	China	*bla*$_{\text{TEM-1}}$, *qepA*, and *aadA1*	–
pWE_H_2	*Escherichia coli*	MW574948.1	54,920	2018	Sweden	*bla*$_{\text{CTX-M-32}}$, *qnrS1*, *tet*(A), *strAB*, and *sul2*	–
pYLPI24b	*Escherichia coli*	CP074014.1	38,883	2018	Cuba	*aadA1*, and *lnu*(F)	–
p30COLEC-2	*Escherichia coli*	CP070932.1	34,313	2019	Singapore	*catB4*	–
pCUVET19-1426.1	*Escherichia coli*	CP115366.1	123,620	2019	Thailand	*bla*$_{\text{CTX-M-55}}$, *bla*$_{\text{TEM-1}}$ (x2), *qnrS1*, *tet*(A), *aadA1*, *aadA2*, *dfrA12*, *sul2*, *sul3*, *cmlA1*, *floR*, and *mef*(B)	This study
pDY612-2	*Escherichia coli*	CP047471.1	75,321	2019	China	*bla*$_{\text{TEM-1}}$, *aadA1*, *aadA5*, *dfrA17*, *catB4*, *floR*, and *cfr*	–
pK342-KPC	*Klebsiella michiganensis*	OM938015.1	209,340	2019	China	*bla*$_{\text{KPC-2}}$, and *qnrS1*	–
pLR-33_1	*Escherichia coli*	AP027524.1	50,339	2019	Ecuador	*mcr-1*	–
pPG20180170.1-IncN	*Escherichia coli*	CP043188.1	42,596	2019	Canada	*bla*$_{\text{CTX-M-1}}$	–
pPG20180171.1-IncN	*Escherichia coli*	CP043186.1	42,591	2019	Canada	*bla*$_{\text{CTX-M-1}}$	–
pPG20180172.1-IncN	*Escherichia coli*	CP043184.1	43,125	2019	Canada	*bla*$_{\text{CTX-M-1}}$	–
pPSJ-07_2	*Escherichia coli*	AP027621.1	51,444	2019	Ecuador	*mcr-1*	–
pPSS-10_4	*Escherichia coli*	AP027689.1	51,832	2019	Ecuador	*mcr-1*	–
pS166-1.4	*Klebsiella pneumoniae*	CP063949.1	83,704	2019	China	*bla*$_{\text{CTX-M-15}}$, *bla*$_{\text{TEM-1}}$, *qepA*, and *aadA1*	–
pYUSHP2-3	*Enterobacter hormaechei*	CP073774.1	44,286	2019	China	*bla*$_{\text{TEM-1}}$, *strAB*, and *rmtB*	–
pC5_41608	*Klebsiella pneumoniae*	MF953243.1	41,608	2020	USA	*bla*$_{\text{CTX-M-1}}$, and *mph*(A)	–

Plasmid name	Bacterial species	GenBank accession no.	Size (bp)	Year	Country	Resistance gene	Reference
pHNBS17e	*Escherichia coli*	MK416152.1	75,167	2020	China	*bla*$_{\text{CTX-M-55}}$, *bla*$_{\text{TEM-141}}$, and *fosA*	–
pTL-1	*Salmonella* sp.	CP104860.1	45,301	2020	China	*bla*$_{\text{TEM-1}}$, and *tet*(A)	–
pW17-3-a	*Moellerella wisconsensis*	CP093261.1	102,837	2021	Poland	*bla*$_{\text{CTX-M-32}}$, *aadA1*, *aph(3')-Ia*, *dfrA1*, *sul1*, and *lnu*(F)	–
pW49-2-b	*Escherichia marmotae*	CP093241.1	54,584	2021	Poland	*bla*$_{\text{CTX-M-32}}$, *qnrS1*, *tet*(A), *strAB*, and *sul2*	–
pSCLC3-1_5	*Klebsiella pneumoniae*	CP113199.1	36,155	2022	China	*aph(3')-IIa*	–
pSCLC4-1_5	*Klebsiella pneumoniae*	CP113206.1	36,155	2022	China	*aph(3')-IIa*	–
pSYCC22-1_4	*Klebsiella pneumoniae*	CP113214.1	96,379	2022	China	*bla*$_{\text{OXA-181}}$, *tet*(A), *aadA1*, *aadA15*, *aph(3')-IIa*, *dfrA14*, *sul1*, *ere*(A), *arr-2*, and *arr-3*	–
pSYCC6-1_5	*Klebsiella pneumoniae*	CP113225.1	42,644	2022	China	*aph(3')-IIa*	–
pRYC492-53	*Klebsiella pneumoniae*	CP127840.1	53,326	1970/1976	Spain	*bla*$_{\text{TEM-1B}}$, *strAB*, *aph(3')-IIa*, *aph(6)-Ic*	–
1205p3	*Shigella flexneri*	CP012143.1	42,002	ND	ND	*qnrS1*, and *aac(3)-IId*	–
p1514kTc1_N_I1_ST113	*Escherichia coli*	MW800641.1	129,471	ND	ND	*bla*$_{\text{CTX-M-8}}$, *bla*$_{\text{TEM-1}}$, *strAB*, and *sul2*	–
p160070-CTXM	*Klebsiella pneumoniae*	MG288677.1	66,643	ND	China	*bla*$_{\text{CTX-M-65}}$, *aadA1*, *fosA*, and *lnu*(F)	–
P18-9079S	*Escherichia coli*	MW349106.1	42,407	ND	USA	*bla*$_{\text{CTX-M-1}}$	–
pESBL-blaCTXM-55	*Escherichia coli*	OQ423470.1	116,909	ND	ND	*bla*$_{\text{CTX-M-55}}$, *bla*$_{\text{TEM-141}}$, *aadA1*, *aph(3')-IIa*, *sul2*, *floR*, and *fosA*	–
pESBL3231-IncN	*Escherichia coli*	MW390537.1	43,277	ND	ND	*bla*$_{\text{SHV-12}}$, *bla*$_{\text{TEM-105}}$, *aadA1*, and *dfrA1*	–
pF404-4	*Escherichia coli*	OY754368.1	43,051	ND	ND	*tet*(A)	–
pHHA45	*Escherichia coli*	JX065630.1	39,510	ND	ND	*bla*$_{\text{CTX-M-1}}$, and *mph*(A)	(2)
pKV7.VI	*Escherichia coli*	LT795508.1	39,744	ND	ND	*bla*$_{\text{CTX-M-1}}$, and *mph*(A)	–
R46	*Salmonella enterica*	AY046276	50,969	ND	ND	*bla*$_{\text{OXA-2}}$, *tet*(C), *aadA1b*, and *sul1*	(3)
pMH13-009N_1	*Proteus mirabilis*	AP018566.2	72,564	ND	Vietnam	*bla*$_{\text{NDM-1}}$, *bla*$_{\text{TEM-1}}$, *qepA*, *tet*(A), *aadA1*, and *rmtB*	–
pMH16-367M_1	*Morganella morganii*	AP018565.2	73,129	ND	Vietnam	*bla*$_{\text{NDM-1}}$, *bla*$_{\text{TEM-1}}$, *qepA*, *tet*(A), *aadA1*, and *rmtB*	–
pNUITM-VR1_4	*Raoultella ornithinolytica*	AP025013.1	107,444	ND	ND	*bla*$_{\text{NDM-1}}$, *bla*$_{\text{TEM-1}}$, *qepA*, *qnrS1*, *tet*(A), *strAB*, *aadA1*, *rmtB*, and *sul2*	–
pRSB201	Uncultured bacterium	JN102341.1	56,488	ND	ND	*bla*$_{\text{TEM-1}}$, *tet*(A), *aph(3'')-Ib*, *aph(6)-Id*, and *aadA1*	(4)
pRSB203	Uncultured bacterium	JN102342.1	42,875	ND	ND	*tet*(B), *aph(3'')-Ib*, and *aph(6)-Id*	(4)
pT199A	*Escherichia coli*	MW298656.1	42,578	ND	ND	*bla*$_{\text{CTX-M-1}}$	–
pVQS1	*Salmonella enterica*	JQ609357.1	40,995	ND	ND	*bla*$_{\text{TEM-1}}$, and *qnrS1*	(5)
unnamed4	*Citrobacter freundii*	MT745953.1	51,531	ND	ND	*tet*(X)	–
unnamed5	*Salmonella enterica*	MW349107.1	42,407	ND	USA	*bla*$_{\text{CTX-M-1}}$	–

Plasmid name	Bacterial species	GenBank accession no.	Size (bp)	Year	Country	Resistance gene	Reference
p100_NDM5_IncN	*Escherichia coli*	MT199177.1	141,007	ND	Italy	bla_{NDM-5}, $bla_{CTX-M-1}$, *tet*(A), *aadA1*, *aadA2*, *aac(3)-IIa*, *dfrA12*, *sul1*, and *mph*(A)	–
pSl1	*Serratia liquefaciens*	CP048785.1	23,411	ND	Germany	*qnrS1*	–
pZPK-H11	*Escherichia coli*	LR999863.1	72,412	ND	Finland	$bla_{CTX-M-1}$, *qnrS1*, *aadA1*, and *lnu*(F)	(6)

ND, not determined.; –, the plasmid was not published in original research articles.

References

1. Clinical and Laboratory Standards Institute. 2024. Performance standards for antimicrobial disk and dilution susceptibility tests for bacteria isolated from animals. 7th ed. CLSI document VET01S. Clinical and Laboratory Standards Institute, Wayne, PA.

2. Dolejska M, Villa L, Hasman H, Hansen L, Carattoli A. 2013. Characterization of IncN plasmids carrying *bla*$_{\text{CTX-M-1}}$ and *qnr* genes in *Escherichia coli* and *Salmonella* from animals, the environment and humans. J Antimicrob Chemother 68:333-339. https://doi.org/10.1093/jac/dks387

3. Hall RM, Vockler C. 1987. The region of the IncN plasmid R46 coding for resistance to β-lactam antibiotics, streptomycin/spectinomycin and sulphonamides is closely related to antibiotic resistance segments found In IncW plasmids and in Tn*21*-like transposons. Nucleic Acids Res 15:7491-7501. https://doi.org/10.1093/nar/15.18.7491

4. Eikmeyer F, Hadiati A, Szczepanowski R, Wibberg D, Schneiker-Bekel S, Rogers LM, Brown CJ, Top EM, Pühler A, Schlüter A. 2012. The complete genome sequences of four new IncN plasmids from wastewater treatment plant effluent provide new insights into IncN plasmid diversity and evolution. Plasmid. 68:13-24. https://doi.org/10.1016/j.plasmid.2012.01.011

5. Karczmarczyk M, Stephan R, Hächler H, Fanning S. 2012. Complete nucleotide sequence of pVQS1 containing a quinolone resistance determinant from *Salmonella enterica* serovar Virchow associated with foreign travel. J Antimicrob Chemother 67:1861-1864. https://doi.org/10.1093/jac/dks158

6. Kurittu P, Khakipoor B, Brouwer MS, Heikinheimo A. 2021. Plasmids conferring resistance to extended-spectrum beta-lactamases including a rare IncN+IncR multireplicon carrying *bla*$_{\text{CTX-M-1}}$ in *Escherichia coli* recovered from migrating barnacle geese (*Branta leucopsis*). Open Res Eur 1:46. https://doi.org/10.12688/openreseurope.13529.1
